# The Anticonvulsant Effect of Hydroethanolic Leaf Extract of *Calotropis procera* (Ait) R. Br. (Apocynaceae)

**DOI:** 10.1155/2021/5566890

**Published:** 2021-06-26

**Authors:** Ernest Obese, Robert Peter Biney, Isaac Tabiri Henneh, Emmanuel Awintiig Adakudugu, Daniel Anokwah, Lovia Serwaa Agyemang, Eric Woode, Elvis Ofori Ameyaw

**Affiliations:** ^1^School of Pharmacy and Pharmaceutical Sciences, College of Health and Allied Sciences, University of Cape Coast, Cape Coast, Ghana; ^2^Department of Biomedical Sciences, School of Allied Health Sciences, College of Health and Allied Sciences, University of Cape Coast, Cape Coast, Ghana; ^3^Department of Pharmacology, Faculty of Pharmacy and Pharmaceutical Sciences, University of Allied Health Sciences, Ho, Ghana

## Abstract

A number of currently used drugs have been obtained from medicinal plants which are a major source of drugs. These drugs are either used in their pure form or modified to a semisynthetic drug. Drug discovery through natural product research has been fruitful over the years. Traditionally, *Calotropis procera* is used extensively in the management of epilepsy. This study is conducted to explore the anticonvulsant effect of a hydroethanolic leaf extract of *Calotropis procera* (CPE) in murine models. This effect was evaluated using picrotoxin-induced convulsions, strychnine-induced convulsions, and isoniazid- and pilocarpine-induced *status epilepticus* in mice of both sexes. The results showed that CPE (100-300 mg/kg) exhibited an anticonvulsant effect against strychnine-induced clonic seizures by significantly reducing the duration (*p* = 0.0068) and frequency (*p* = 0.0016) of convulsions. The extract (100-300 mg/kg) caused a profound dose-dependent delay in the onset of clonic convulsions induced by picrotoxin (*p* < 0.0001) and tonic convulsions (*p* < 0.0001) in mice. The duration of convulsions was reduced significantly also for both clonic and tonic (*p* < 0.0001) seizures as well. CPE (100-300 mg/kg), showed a profound anticonvulsant effect and reduced mortality in the pilocarpine-induced convulsions. ED_50_ (~0.1007) determined demonstrated that the extract was less potent than diazepam in reducing the duration and onset of convulsions but had comparable efficacies. Flumazenil—a GABA_A_ receptor antagonist—did not reverse the onset or duration of convulsions produced by the extract in the picrotoxin-induced seizure model. In isoniazid-induced seizure, CPE (300 mg kg^1^, *p.o.*) significantly (*p* < 0.001) delayed the onset of seizure in mice and prolonged latency to death in animals. Overall, the hydroethanolic leaf extract of *Calotropis procera* possesses anticonvulsant properties.

## 1. Introduction

Epilepsy is a neurological condition that affects people of various age categories and is marked by excessive or abnormal electric activity occurring in either a portion or all of the brain [1]. Seizures that can happen spontaneously and repeatedly are known as outward signs of epilepsy. The seizures may well be caused by various conditions such as stroke, brain tumour, head injury, or infection of the central nervous system [1]. It is projected that about fifty million individuals worldwide currently live with epilepsy and the disorder is responsible for one percent of the global burden of diseases [2] and is typically higher in low- and middle-income countries [3].

Despite the broad range of pharmacological agents approved for patients with epilepsy, many people are still nonresponsive or refractory to antiepileptic drug therapy, rendering pharmacoresistance one of the most significant clinical issues in the management of the disease [3]. Also, these treatments only resolve epilepsy symptoms and do not properly prevent the occurrence of seizures or permanently stop seizures [4]. New, better, and safer antiepileptic drugs (AEDs) with enhanced clinical profiles need to be developed. Traditional medicine has contributed greatly to the discovery of many drugs, including morphine, digoxin, quinine, and atropine [5].

Plant extracts are some of the most appealing sources of fresh drugs, and promising outcomes have been shown for epilepsy therapy. Examples include *Antiaris toxicaria*, *Pseudospondias microcarpa*, and *Mallotus oppositifolius* [6–8]. Since it is estimated that around 80% of the population in the developing world use herbal remedies for their primary health care needs [9], conditions like epilepsy and pain are usually managed using herbs. Plant products traditionally used in the therapy of epilepsy can serve as a basis for identifying and developing alternative antiepileptics, as they may contain bioactive compounds that can mitigate seizures [10]. *Calotropis procera* is another example of medicinal plants used traditionally in the management of epilepsy [11].

In our laboratory, *Calotropis procera* extract showed a significant central nervous system (CNS) depressant effect and the ability to delay and reduce the frequency of seizures in a primary neuropharmacological screening test. With this, in combination with its conventional use in the management of epilepsy and other CNS disorders [11], the extract's anticonvulsant property can be further assessed to confirm its anticonvulsant qualities. It has also been shown to have significant anti-inflammatory and antinociceptive properties, and since pain and inflammation are acutely manifested in epilepsy [12, 13], these effects may augment the possible anticonvulsant effect of *C. procera* in epilepsy management.

The chemical fingerprint of herbal products is an accepted strategy by the World Health Organisation (WHO) to authenticate and assessment of the quality of medicinal plants [14]. Although there are several analytical techniques for developing fingerprint, High-Performance Liquid Chromatography (HPLC) is the most popular technique which provides a rapid fingerprint for identifying and assessing the quality of medicinal plants [15].

The present study was conducted to develop an HPLC fingerprint and further explore the anticonvulsant potentials of the hydroalcoholic extract of the leaves of *C. procera* by employing acute seizure models using agents such as picrotoxin and strychnine. The pilocarpine (a human temporal lobe epilepsy model) and isoniazid models of *status epilepticus* were also employed in characterizing the anticonvulsant effect of the extract. The possible involvement of the benzodiazepine/gamma amino butyric acid (GABA) receptor complex in the mechanism of action of the extract was also investigated.

## 2. Materials and Methods

### 2.1. Plant Collection and Extraction

Fresh leaves of *Calotropis procera* were collected from Iture (5° 05′ 54.6^″^ N, 1°18′ 48.7^″^ W), a town near the University of Cape Coast (UCC), from August to December 2015. Leaves of *C*. *procera* were authenticated by Mr. Fynn, a botanist, at the School of Biological Sciences Herbarium, University of Cape Coast, and the voucher number UCC/SBSH/15/M044 was assigned. A hydroalcoholic extract was produced as previously described [12, 16]. The leaves were air-dried for fourteen days and powdered. An amount of 200 g of the powdered leaves was soaked with 2 L of 70% ethanol for 72 h, filtered, and concentrated using a rotary evaporator (Rotavapor R-215, BÜCHI Labortechnik AG, Flawil, Switzerland) under reduced pressure and temperature (50°C). The extract, CPE (*Calotropis procera* extract), was further dried and preserved in a desiccator containing activated silica until it was ready for use. The yield obtained was 4.8% *w*/*w*. The extract was reconstituted for use in the experiments by gently triturating to prepare a solution of it with distilled water as the vehicle.

### 2.2. HPLC Fingerprint

The freshly prepared extract (200 mg) was reconstituted in 10 mL of 70% *v*/*v* ethanol to obtain a stock solution (2% *w*/*v*). About 2 mL of the solution was filtered through a 0.45 *μ*m membrane filter into the sample vial.

The HPLC analysis of the extract was carried out on a PerkinElmer Flexar system consisting of a binary pump, Photo Diode Array detector, autosampler, and online degasser. The separation was carried out on the Zorbax 300SB-C18 analytical column (4.6 × 250 mm, 5 *μ*m) from Agilent Technologies with an injection volume of 20 *μ*L and an acquisition wavelength of 280 nm. The mobile phase comprising 0.05% trifluoroacetic acid (A) and methanol (B) was employed with gradient elution at a flow rate of 1 mL/min at ambient temperature. The gradient elution was programmed as follows: 0-3 min, 15% B; 3-23 min, 90% B; 23-26 min, 90% B; 26-27 min, 15% B; and 27-32 min, 15% B [16].

### 2.3. Animals

ICR mice of both sexes (20-25 g) were acquired from the Noguchi Memorial Institute for Medical Research and cared for in the Animal House Department. They were kept in the animal house of the School of Biological Sciences, University of Cape Coast (UCC) for seven days to acclimatize before the experiments. The animals were housed in cages (34 × 47 × 18 cm^3^) with softwood shavings as bedding and were maintained at a 12 h light-dark cycle. They had free access to food and water. The studies conducted were following the National Institute of Health (NIH) Guidelines for Care and Use of Laboratory Animals with approval from the Department of Pharmacology Ethics Committee.

### 2.4. Drugs and Chemicals

Diazepam (DZP) (>99% (HPLC), solid), picrotoxin (PTX) (>97.5% (HPLC), solid), strychnine (STR) (98% powder, solid), isoniazid (analytical standard, ≥99% (TLC)), and pilocarpine (99% (titration), powder) were purchased from Sigma-Aldrich Chemie GmbH (Eschenstrasse) Germany. Flumazenil (FLU) was purchased from Roche, (Basel) Switzerland (>99% (HPLC), solid).

### 2.5. Strychnine-Induced Seizure Test

The experimental model used was as described previously [8, 17]. Briefly, strychnine seizures were induced in male ICR mice (*n* = 7) by the injection of strychnine nitrate, (0.5 mg/kg i.p.) 1 h after administration of the extract (30-300 mg/kg, *p.o.*) or 30 min after diazepam (0.1–1.0 mg/kg, i.p.) administration. The frequency, duration, and also latency to myoclonic jerks were recorded for extract-treated groups and the diazepam group compared to the saline-treated animals (control). These were observed through video recording (Sony-Handycam, model: HDRCX675/B, Tokyo, Japan) for 30 min. Animals were tracked using the behavioural analysis software, JWatcher™ version 1.0 (University of California, Los Angeles, USA, and Macquarie University, Sydney, Australia. Available at http://www.jwatcher.ucla.edu).

### 2.6. Pilocarpine-Induced *Status Epilepticus*

This experiment followed a procedure previously described [8, 18]. Pilocarpine (300 mg/kg, i.p.) was administered to induce seizures by injection to ICR mice (*n* = 7). CPE (30-300 mg/kg, *p.o.*) or diazepam (0.1-1.0 mg/kg, i.p.) or normal saline (10 mL kg^−1^*p.o.*) was administered 1 h after oral or 30 min after i.p. administration before induction of seizures. N-Butyl-bromide hyoscine (1 mg/kg, i.p.) was administered 30 min prior to induction to counter the peripheral autonomic effects produced by pilocarpine. After the injection of pilocarpine, the animals were placed separately into the transparent Plexiglas testing chamber and the latency to and duration of clonic tonic seizures were observed through video recordings and tracked as outlined in Section 2.5. Dose-response curves were plotted and ED_50_ (a measure of anticonvulsant potency) and *E*_max_ (a measure of efficacy) were obtained from the curves.

### 2.7. Isoniazid-Induced Seizure Model

The method was as previously described [19]. Animals were adapted to the chamber for 1 h before receiving treatment with either CPE (30–300 mg/kg, *p.o.*), diazepam (0.1-1 mg/kg i.p.), or saline (10 mL/kg, i.p.). Seizures were induced with isoniazid (INH) (300 mg/kg, *p.o*.) 30 min or 1 h after i.p. or *p.o.* treatment, respectively. Animals were observed and tracked as previously described in Section 2.5.

### 2.8. Picrotoxin-Induced Seizure Model

The procedure used was the same as discussed in the pilocarpine seizure test described in Section 2.5 except that seizures induced in mice (*n* = 7) were by administration of picrotoxin 3 mg/kg, i.p. [6] 30 min and 1 h after treatment with diazepam (0.1-1 mg/kg, i.p.) and CPE (30–300 mg/kg, *p.o.*), respectively. Animals in the control group received normal saline (10 mL/kg, *p.o*.). The latency to tonic convulsions, latency to myoclonic jerks, and also the frequency and duration of tonic convulsions were recorded from the videos for each mouse as previously described in Section 2.5.

### 2.9. Involvement of the GABAergic System

A selective benzodiazepine receptor antagonist, flumazenil (FLU), was used to investigate the involvement of GABA_A_ receptors in the anticonvulsant mechanism of CPE. Animals (*n* = 7) received CPE (100 mg/kg, *p.o*.), diazepam (0.3 mg/kg, i.p.), flumazenil (2 mg/kg), and normal saline (10 mL/kg, *p.o.*) 30 min before the administration of picrotoxin (3 mg/kg i.p.). The last two groups were given flumazenil (2 mg/kg, i.p.) 5 min before the administration of CPE (100 mg/kg, *p.o*.) or diazepam (0.3 mg/kg, i.p.) and 65 min or 35 min before the injection of picrotoxin (3 mg/kg, i.p.), respectively. The latency to frequency and duration of clonic convulsions were tracked as described in Section 2.5.

### 2.10. Data Analysis

Data from the experiments were presented as the mean ± standard error of mean (S.E.M.). Two-way analysis of variance (ANOVA) was used in analysing time-course curves whiles a one-way analysis of variance was employed in determining differences between treatment groups (areas under the curve). GraphPad Prism version 7.0 (GraphPad Software, San Diego, CA) for Windows was used to perform all statistical analyses with *p* < 0.05 considered statistically significant for all tests.

## 3. Results

### 3.1. HPLC Fingerprint

**Figure 1:**
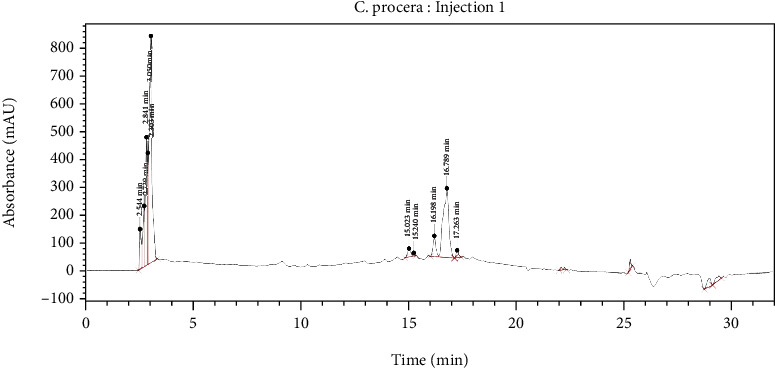
HPLC chromatogram of the 70% hydroethanolic extract of *C. procera* leaves.

The HPLC chromatogram (Figure 1) revealed 10 peaks representing the various constituents in the 70% hydroethanolic extract of *C. procera* leaves. The retention times of the peaks with their respective area and height are represented in Table 1.

### 3.2. Strychnine-Induced Seizures

**Figure 2:**
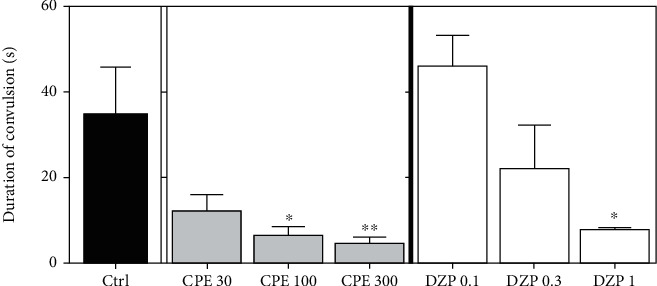
Effect of CPE (30-300 mg/kg, *p.o.*) and diazepam (0.1-1.0 mg/kg, i.p.) on the duration of strychnine-induced clonic seizures in mice. Data are expressed as mean ± S.E.M. (*n* = 7). ^∗∗^*p* < 0.01 and ^∗^*p* < 0.05 (one-way ANOVA followed by Tukey's *post hoc* test).

Figure 2 indicates the effects of CPE (30-300 mg/kg, *p.o.*) and diazepam (0.1-1 mg/kg, i.p.) on the duration of clonic convulsions induced by strychnine in mice. The administration of the extract was able to reduce the duration of convulsions as well as reduce the frequency of clonic seizures. However, the extract showed a relatively inadequate ability to delay the onset of these seizures induced by strychnine.

**Figure 3:**
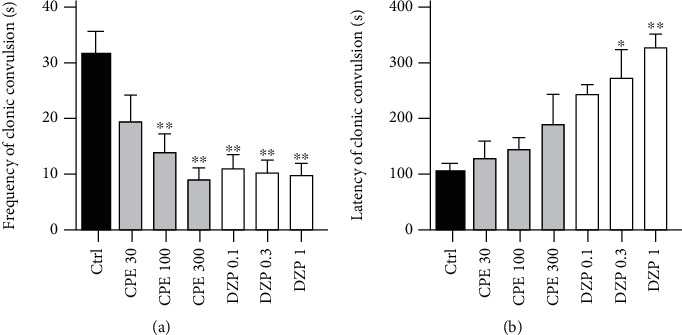
Effect of CPE (30-300 mg/kg, *p.o.*) and diazepam (0.1-1.0 mg/kg, i.p.) on frequency (a) and latency (b) of strychnine-induced clonic seizures in mice. Data are expressed as mean ± S.E.M. (*n* = 7). ^∗∗^*p* < 0.01 and ^∗^*p* < 0.05 (one-way ANOVA followed by Dunnett's *post hoc* test).

One-way ANOVA revealed that the CPE (100-300 mg/kg) exhibited a dose-dependent anticonvulsant effect against strychnine-induced clonic seizures by significantly reducing the duration of convulsions (*p* = 0.0068). Diazepam (1 mg/kg) also significantly reduced the duration of strychnine-induced clonic seizures. The extract (100-300 mg/kg) significantly (*p* = 0.0016) reduced the frequency of clonic convulsions induced by strychnine (Figure 3(a)). However, CPE, at the doses given (30–300 mg/kg) was unable to increase the latency of strychnine-induced clonic convulsions significantly. Diazepam (0.3-1 mg/kg) significantly (*p* = 0.0188) delayed the onset of convulsions (Figure 3(b)), and all doses of diazepam administered significantly reduced the frequency of convulsions (Figure 3(a)).

#### 3.2.1. Pilocarpine-Induced *Status Epilepticus*

**Figure 4:**
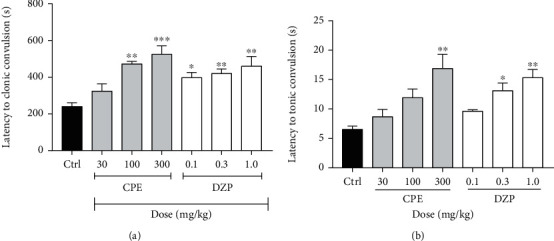
Effect of CPE (30-100 mg/kg, p.o.) and diazepam (0.1-1 mg/kg, i.p.) on the latency to (a) clonic and (b) tonic convulsions in the pilocarpine-induced status epilepticus in mice. Data is presented as mean ± S.E.M. (*n* = 7); ^∗∗∗^*p* < 0.001; ^∗∗^*p* < 0.01, and ^∗^*p* < 0.05 compared to the vehicle-treated group (one-way ANOVA followed by Dunnett's *post hoc* test).

**Figure 5:**
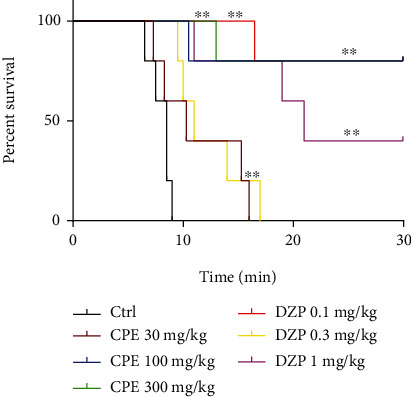
Percentage survival of mice for extract (30-300 mg/kg) and diazepam (0.1-1 mg/kg, i.p.). Each point is the mean ± S.E.M. of 7 animals.

**Figure 6:**
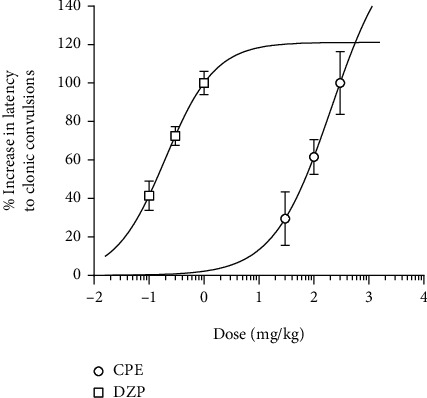
Dose-response curve for the anticonvulsant activity induced by the administration of CPE (30-100 mg/kg, *p.o.*) and diazepam (0.1-1 mg/kg, i.p.) in the pilocarpine-induced seizure test in mice.

One-way ANOVA showed that CPE dose-dependently delayed the onset of clonic (*p* = 0.0001) (Figure 4(a)) and tonic convulsions (*p* = 0.0001) (Figure 4(b)). Diazepam, the reference anticonvulsant agent (DZP 0.1-1.0 mg/kg), showed similar effects as the extract by increasing the latencies to clonic and tonic convulsions. The oral dose of CPE (100-300 mg/kg) showed a profound anticonvulsant effect by protecting the animals against death, which was caused by the convulsions induced by pilocarpine (Figure 5) (hazard ratio = 0.1819, *p* < 0.0021). ED_50_ (~0.1007) and *E*_max_ values calculated from the dose-response curves (Figure 6) demonstrated that the extract was less potent than diazepam in reducing the duration of convulsions and delaying the onset of convulsions, but their efficacies were comparable.

#### 3.2.2. Isoniazid-Induced Seizure Model

**Figure 7:**
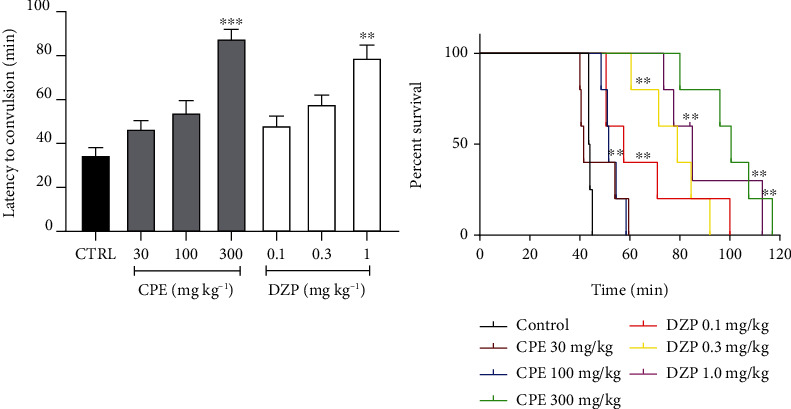
Effect of extract (CPE 30-300 mg/kg, *p.o*.) and diazepam (DZP 0.1-1.0 mgkg^−1^, i.p.) on the latency to convulsion and survival time curve of isoniazid-induced epilepsy in mice. Data is presented as mean ± S.E.M. (*n* = 7); ^∗∗∗^*p* < 0.001; ^∗∗^*p* < 0.01 compared to the vehicle-treated group (one-way ANOVA followed by Dunnett's *post hoc* test).

The extract, CPE, significantly prolonged the onset of and survival in seizures induced by isoniazid in mice. Analysis of the results showed that CPE (300 mg kg^1^, *p.o.*) delayed the onset of the seizure (*p* < 0.001) as shown in Figure 7(a). Diazepam showed similar effects with the highest dose (1 mg/kg significant at *p* < 0.01). The extract CPE (100-300 mg/kg) again exhibited anticonvulsant action by protecting (prolonged latency to death) animals against death, which was caused by the convulsions induced by isoniazid (Figure 7(b)).

#### 3.2.3. Picrotoxin-Induced Seizures

**Figure 8:**
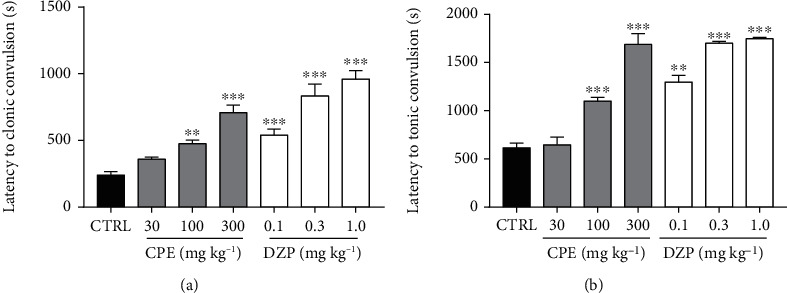
Effect of CPE (30-300 mg/kg*, p.o*.) on the latency to (a) clonic convulsion and (b) tonic convulsions in the picrotoxin-induced seizure test in mice. Data are presented as mean ± S.E.M. (*n* = 7); ^∗∗∗^*p* < 0.001; ^∗∗^*p* < 0.01; compared to the control group (one-way ANOVA followed by Dunnett's *post hoc* test).

**Figure 9:**
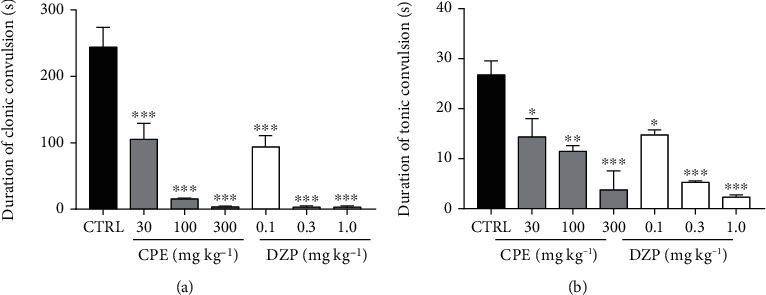
Effect of CPE (30-300 mg/kg, *p.o*.) on the duration of convulsions in the picrotoxin-induced seizure test in mice. Data are presented as mean ± S.E.M. (*n* = 7); ^∗∗∗^*p* < 0.001; ^∗∗^*p* < 0.01; ^∗^*p* < 0.05 compared to the control group (one-way ANOVA followed by Dunnett's *post hoc* test).

**Figure 10:**
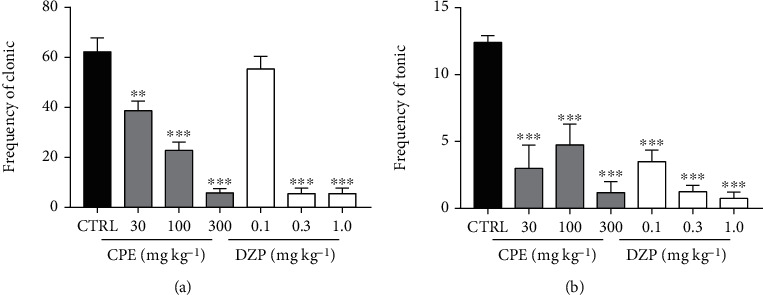
Effect of CPE (30-300 mg/kg, *p.o*.) on the frequency of convulsions in the picrotoxin-induced seizure test in mice. Data are presented as mean ± S.E.M. (*n* = 7); ^∗∗∗^*p* < 0.001 and ^∗∗^*p* < 0.01 compared to the control group (one-way ANOVA followed by Dunnett's *post hoc* test).

The extract-treated groups exhibited a significant anticonvulsant effect in this model. CPE (100–300 mg/kg) caused a profound dose-dependent delay in the onset of clonic convulsions (*p* < 0.0001) (Figure 8(a)) and tonic convulsions in mice (*p* < 0.0001) (Figure 8(b)). The extract also decreased the duration of convulsions significantly in clonic (*p* < 0.0001) (Figure 9(a)) and tonic (*p* < 0.0001) convulsions (Figure 9(b)). Diazepam (0.1–1.0 mg/kg), the reference anticonvulsant, showed similar results as the extract by increasing the latencies to clonic and tonic convulsions and the duration of convulsions. The frequencies of convulsions were also reduced significantly in both clonic (*p* < 0.0001) and tonic (*p* < 0.0001) seizures (Figure 10).

#### 3.2.4. Involvement of GABAergic Mechanism

**Figure 11:**
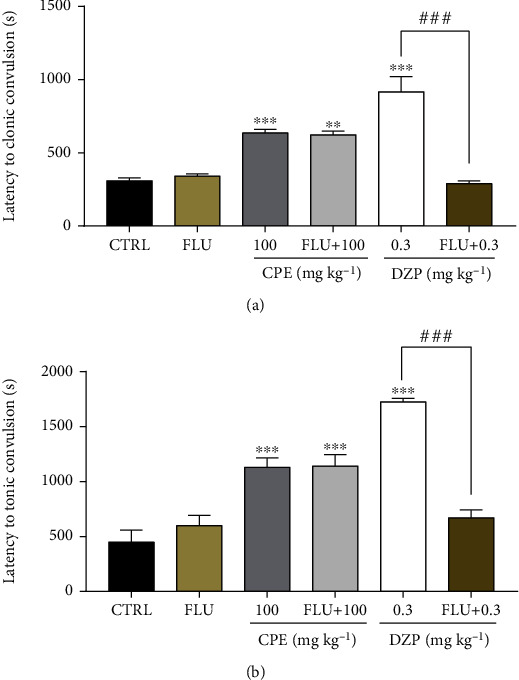
Effect of flumazenil on the anticonvulsant effect (latency to convulsion) of CPE (100 mg/kg, *p.o*.). (a) Latency to clonic and (b) latency to tonic convulsion in the picrotoxin-induced seizure test in mice. Data are presented as mean ± S.E.M. (*n* = 7); ^∗∗∗∗^*p* < 0.0001; ^∗∗∗^*p* < 0.001, and ^∗∗^*p* < 0.01; compared to the vehicle-treated group (One-way ANOVA followed by Dunnett's *post hoc* test). ^###^*p* < 0.001 compared to the diazepam-treated group.

**Figure 12:**
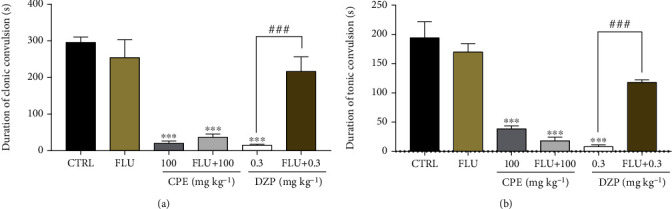
Evaluation of GABAergic involvement on the duration of (a) clonic and (b) tonic seizures in the picrotoxin-induced seizure test in mice treated with CPE (100 mg/kg, *p.o*.). Data are presented as mean ± S.E.M. (*n* = 7); ^∗∗∗∗^*p* < 0.0001; ^∗∗∗^*p* < 0.001, compared to the vehicle-treated group (one-way ANOVA followed by Dunnett's *post hoc* test). ^***###***^*p* < 0.001 compared to the diazepam-treated group.

**Figure 13:**
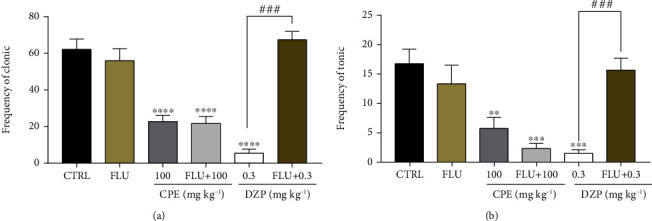
Evaluation of GABAergic involvement on the frequency of (a) clonic and (b) tonic seizures in the picrotoxin-induced seizure test in mice treated with CPE (100 mg/kg, *p.o*.). Data are presented as mean ± S.E.M. (*n* = 7); ^∗∗∗∗^*p* < 0.0001; ^∗∗∗^*p* < 0.001; compared to the vehicle-treated group (one-way ANOVA followed by Dunnett's post hoc test). ^**###**^*p* < 0.001 compared to the diazepam-treated group.

From the results obtained, CPE alone delayed the onset, duration, and frequency of convulsions just like diazepam alone (Figures 11–13). Flumazenil alone (2 mg/kg, i.p.) did not alter the onset or duration of convulsions. Pretreatment with flumazenil could not inhibit the anticonvulsant effect of the extract but significantly reversed the onset of clonic (*p* < 0.0001) and tonic (*p* < 0.0001) convulsions; the duration of clonic (*p* < 0.0001) and tonic (*p* < 0.0001); and frequency of clonic (*p* < 0.0001) and tonic (*p* < 0.0001) convulsion effect of diazepam in the picrotoxin-induced seizure model.

## 4. Discussion

To ensure correct identification and authentication of the C. procera leaf extract, an HPLC fingerprint was developed. HPLC fingerprint analysis of plant extracts provides information on the retention time of the constituents in a plant extract as a quality control method for identifying and assessing the stability of the plant extract [20]. The chromatogram revealed the presence of five (5) peaks showing the retention time of 2.5-3.1 minutes suggesting the presence of highly polar constituents. Five (5) peaks showed between 15 and 17.3 minutes suggesting the presence of relatively nonpolar constituents in the hydroethanolic extract of *C. procera*. This was similar to other earlier reports [16]. The peaks at retention times of 3.050 and 17.263 could be attributed to the presence of catechol and calotoxin, respectively, as reported [15]. The HPLC fingerprint provided in this work would be beneficial for identifying and assessing the quality of the 70% hydroethanolic extract of the leaves of *C. procera*. Again the results from the HPLC forms the basis for the isolation and characterization of compounds from the extract in future studies.

It is clear from the studies conducted that oral administration of *Calotropis procera* leaf extract mitigates convulsions in both generalized and acute seizure models—pentylenetetrazole, picrotoxin, and strychnine-induced seizure tests, respectively, and partial seizure models; the pilocarpine-induced Status epilepticus and the isoniazid-induced seizures.

The glycine receptor is liable for the regulation of strong inhibitory neurotransmission in the mature central nervous system [21], which makes this receptor a prospective target for antiepileptic drugs [22, 23]. Strychnine induces seizures by blocking the activity of strychnine-sensitive glycine receptors and increased postsynaptic excitability and sustained action in the brainstem and spinal cord [23, 24]. Since the extract decreased the frequency and duration of convulsions caused by strychnine, a possibility of the extract interacting with glycine receptors/pathways is conceivable. The extract may contain bioactive compounds that activate glycinergic inhibitory neurotransmission.

An animal model of intractable epilepsy is the systemic administration of pilocarpine, a nonselective muscarinic agonist [8, 18, 25–27]. Histological studies have shown that this model has important similarities to temporal lobe epilepsy in humans, and thus, in this model, successful drugs are possible candidates for the treatment of temporal lobe epilepsy [8, 28–31]. The extract was able to protect the animals from death from acute convulsions described as status epilepticus induced by pilocarpine. Consequently, in the treatment of temporal lobe epilepsy and/or other partial seizures, the extract may be of possible use. This is evidenced in the survival curve drawn and the hazard ratio calculated. The lower the hazard ratio, the better the treatment as there is a low risk of death compared to an untreated population [32].

Alkaloids, saponins, and sterols, which are identified secondary metabolites in the plant, may be responsible for the anticonvulsant effect of the extract. [12].

Status epilepticus by isoniazid is related to the inhibition of glutamate decarboxylase (GAD), an enzyme required for GABA synthesis [33–36]. Again, decreased levels of GABA in the brain are associated with ongoing seizures seen in animals exposed to high amounts of isoniazid [35]. The depletion of pyridoxine caused using INH leads to decreased development of GABA, as it is normally a result of a decarboxylation reaction that is based on pyridoxine. GABA deficiency can therefore present itself as seizures, especially in the acute toxicity environment [8].

Diazepam was only effective at its highest dose on the latency to death. The variability and immediate availability of the amount of INH ingested are also the key drawbacks to the effectiveness of DZP in the treatment of isoniazid toxicity clinically. [8, 37]. Again, previous studies revealed that anticonvulsant agents do not depend on the prevention of convulsion but on their ability to prolong the latency to seizures [38]. Moreover, compounds that only delay the latency to convulsions block the spread of seizures in an epileptic brain.

Picrotoxin is a GABA_A_ receptor antagonist [39]. Picrotoxin confers its convulsive activity by blocking the chloride ion channel linked to the GABA_A_ receptor, which normally unlocks to allow increased chloride ion conductance into the brain cells after GABA_A_ receptor activation by gamma-aminobutyric acid [40–42]. GABAergic ionotropic receptors can mediate both pre-and postsynaptic inhibition. Presynaptic inhibition mediated by GABA often leads to inhibition of neurotransmitter release from the excitatory arm [43]. The extract, being effective in the picrotoxin-induced seizure test, points to action on GABA-mediated neurotransmission.

Many barbiturates and benzodiazepines generally potentiate the inhibitory action of GABA_A_ receptors, reducing neuronal excitability and increasing the threshold for convulsions [44]. Since CPE displayed effectiveness in both pentylenetetrazole-induced seizures and picrotoxin-induced seizures, a possible association with GABAergic mechanisms was investigated using flumazenil, a benzodiazepine receptor antagonist [45], using the picrotoxin-induced seizure test. However, in this test, flumazenil was unable to reverse the antiseizure effect characterised by CPE and this indicates a potentially different or complex mechanism involved in the attenuation of convulsion by the *Calotropis procera* extract.

These results are consistent with studies conducted previously on the aqueous extract of leaves of *Calotropis procera* in pentylenetetrazole-induced seizures [46]. The aqueous extract increased the onset and duration of convulsions but showed no change in the levels of GABA. This provides further evidence that the mechanism by which the plant exerts its anticonvulsant action may be through a different pathway.

The models employed in this study provide a very good basis for further studies on *C. procera* as an antiepileptic agent. Because compounds were not isolated in this study from the crude extract, the mechanism (s) by which the extract produces its effects cannot be attributed to a specific compound in the plant. Subsequent studies may seek to isolate bioactive substances from the crude extract and determine the precise receptor interactions of these isolates in their anticonvulsant activity.

## 5. Conclusion

These findings demonstrated an anticonvulsant effect of the extract. CPE activation of the GABAergic system may be absent but may act through glycinergic systems. The anticonvulsant effect exhibited makes the extract a likely therapeutic potential for both generalised and partial seizure events.

## Figures and Tables

**Table 1:** Peaks representing various constituents in CPE.

Peak #	RT (min)	Area	Height
1	2.544	816,223.3	144,908.7
2	2.739	1,319,146.6	219,099.5
3	2.841	2,268,039.8	460,169.7
4	2.903	893,419.5	399,447.2
5	3.050	7,357,355.7	811,648.1
6	15.023	305,158.0	30,711.5
7	15.240	56,255.6	10,796.5
8	16.198	679,475.5	73,862.5
9	16.789	4,618,429.7	249,179.4
10	17.263	207,809.2	26,543.7

## Data Availability

The data in support of the findings in this study may be requested and obtained from the corresponding author.
